# Alzheimer disease combination therapy: learning from the HIV drug development cascade

**DOI:** 10.1002/alz.090075

**Published:** 2025-01-09

**Authors:** Bernadeth Lyn C Piamonte, Eric Dinnerstein

**Affiliations:** ^1^ Maine Medical Center, Portland, ME USA

## Abstract

**Background:**

Once an incurable deadly disease of urgent public health concern, human immunodeficiency virus (HIV) infection has now evolved into a chronic condition largely manageable with combination therapy as the treatment standard. In comparison, Alzheimer disease (AD) is a fatal illness that continues to pose epidemiologic and socioeconomic challenges not only to persons and families affected by it but also to the healthcare system as a whole. With the recent approval of multi‐targeted therapies in AD and numerous clinical trials in the pipeline, there is an urgency to search for ways to best maximize their efficacy and utility.

**Method:**

In this review, we compared drug development in HIV and AD and suggested solutions to barriers to promote accelerated combination trials.

**Result:**

The pathophysiology of both diseases is complex and involves multiple steps that may require combination treatment versus monotherapy. Barriers to combination therapy include pharmacological properties between and among drugs affecting their efficacy and safety, issues of high pill burden, conflicts of interest between stakeholders in drug development, the pharmaceutical industry culture of secrecy, and high regulatory hurdles. Pharmacoenhancement, single‐tablet regimens, use of therapeutic drug monitoring and routine medication reconciliation, intense collaboration among stakeholders, and deregulating drug development by giving priority reviews and introducing shorter timelines for approval of hopeful novel drugs and diagnostics are all possible solutions to these barriers.

**Conclusion:**

The history and evolution of combination therapy in HIV offer a good model of accelerated development of successful combination therapy for AD.

